# Cholera Sicca: A Rare and Atypical Presentation of Vibrio cholerae Infection Mimicking Bowel Obstruction

**DOI:** 10.7759/cureus.83343

**Published:** 2025-05-02

**Authors:** Ahmed A Banan, Aseel A Aljunied, Anas M Aljoaid, Khalid W Alhariqi, Abdullmoin M AlQarni

**Affiliations:** 1 Internal Medicine, Al-Noor Specialist Hospital, Makkah, SAU; 2 Infectious Diseases, Al-Noor Specialist Hospital, Makkah, SAU

**Keywords:** acute kidney injury, atypical cholera, cholera sicca, hypovolemic shock, metabolic acidosis, vibrio cholerae

## Abstract

Cholera sicca is a rare and often overlooked variant of cholera, characterized by severe dehydration and circulatory collapse without the hallmark profuse diarrhea. This case report describes a 40-year-old previously healthy male who presented with abdominal distention, vomiting, and minimal bowel movements, initially mimicking bowel obstruction. The patient was hemodynamically unstable, with severe metabolic acidosis (pH 6.95), acute kidney injury (creatinine 610 µmol/L), and hypovolemia shown by a collapsed inferior vena cava on imaging. Stool studies confirmed *Vibrio cholerae* infection, leading to a diagnosis of cholera sicca. Management included aggressive fluid resuscitation, broad-spectrum antibiotics, and supportive care, resulting in clinical improvement. This case highlights the diagnostic challenges of cholera sicca, which can be misdiagnosed due to its atypical presentation. Unlike typical cholera, where profuse diarrhea facilitates early recognition, cholera sicca often presents with minimal or no diarrhea, leading to delayed treatment and higher mortality. Early recognition and aggressive fluid resuscitation are critical to improving outcomes. This report underscores the importance of considering cholera sicca in differential diagnoses, particularly in endemic regions, and emphasizes the need for enhanced diagnostic tools, such as point-of-care ultrasound, to assess volume status. Public health interventions, including improved water sanitation, mass vaccination campaigns, and healthcare worker training, are essential for preventing and managing cholera outbreaks. Cholera sicca is a life-threatening condition that requires prompt recognition and management. This case serves as a reminder of the diverse presentations of cholera and the importance of early intervention to reduce morbidity and mortality in resource-limited settings.

## Introduction

Cholera, caused by the gram-negative bacterium *Vibrio cholerae*, is a rapidly dehydrating diarrheal disease transmitted through contaminated water or food [1]. While typical cholera presents with profuse “rice water” diarrhea, an atypical variant known as cholera sicca is characterized by severe dehydration and circulatory collapse without prominent diarrhea, posing unique diagnostic challenges [2,3]. Despite its clinical significance, cholera sicca remains understudied, with limited epidemiological data or systematic reviews to quantify its rarity or outcomes. Current literature primarily consists of isolated case reports, highlighting a gap in a comprehensive understanding of its prevalence, risk factors, and optimal management [4,5].

The global burden of cholera is substantial, with an estimated 1.3-4 million cases and 21,000-143,000 deaths annually [6]. However, the proportion of cases attributable to cholera sicca is unknown, and its mortality rate may be underestimated due to misdiagnosis. For instance, cases are often mistaken for bowel obstruction or paralytic ileus, delaying life-saving fluid resuscitation [7,8]. This diagnostic ambiguity underscores the need for increased clinical awareness, particularly in endemic regions such as Yemen, where the patient in this report originated amid an ongoing outbreak linked to conflict and poor sanitation [9,10]. In the midst of its ongoing conflict, Yemen saw the greatest cholera outbreak in modern history from 2016 to 2022, with over 2.5 million suspected cases and 4,000 fatalities [11,12]. By the end of 2024, the country reported 249,900 suspected cases of cholera, with 861 associated deaths [11].

This report presents a case of cholera sicca, initially misdiagnosed as bowel obstruction with severe metabolic acidosis, acute kidney injury, and collapsed inferior vena cava on imaging, exemplifying the rapid hemodynamic compromise typical of this cholera sicca variant. By detailing this case, we aim to address the knowledge gap by synthesizing existing evidence on cholera sicca and contrasting it with typical cholera presentations, highlighting diagnostic pitfalls through imaging and laboratory findings that differentiate cholera sicca from other acute abdominal conditions, and emphasizing the urgency of early recognition, as delayed fluid resuscitation, the cornerstone of management, leads to higher mortality in the absence of overt diarrhea [13,14].

## Case presentation

A 40-year-old previously healthy Yemeni male presented to the ED with a two-day history of progressive abdominal distention, generalized cramping abdominal pain, and recurrent vomiting containing food particles. He reported one episode of loose stool followed by constipation but denied hematochezia, hematemesis, or constitutional symptoms. The patient had returned from rural Yemen one week prior, where cholera is endemic due to ongoing outbreaks linked to contaminated water sources. Notably, he had received a single dose of oral cholera vaccine (OCV; likely Shanchol™) three days before symptom onset but could not confirm completion of the recommended two-dose regimen.

On examination, the patient appeared acutely ill with signs of severe dehydration, including sunken eyes and dry mucous membranes. Initial vital signs revealed paradoxical hypertension (172/100 mmHg), likely due to a compensatory catecholamine surge, along with tachycardia (140 bpm), tachypnea (30 breaths/min), and low-grade fever (37.7°C). Abdominal examination demonstrated distention with diffuse tenderness but no peritoneal signs. A digital rectal exam revealed an empty rectum with scant mucus output. The patient was oliguric, producing only 100 mL of urine via Foley catheter over several hours.

**Figure 1:**
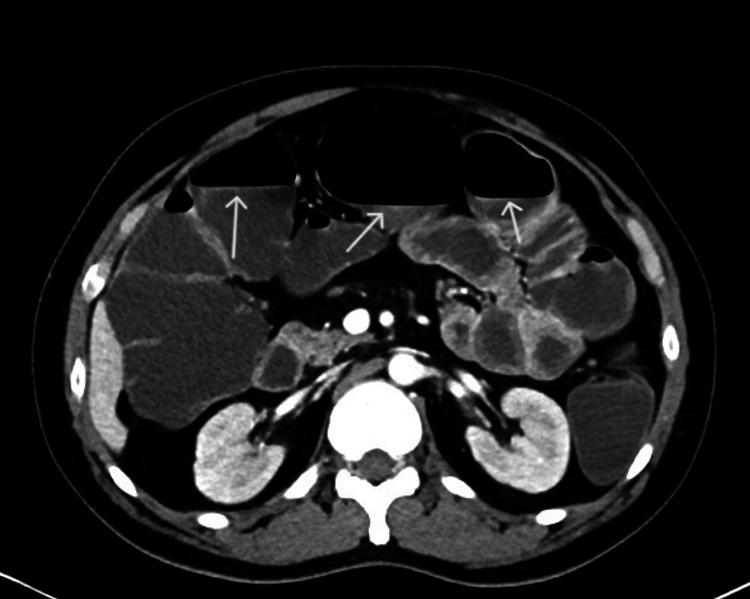
Collapsed inferior vena cava (arrows)

Laboratory findings were remarkable for severe metabolic acidosis (pH 6.95, HCO₃⁻ 7.5 mmol/L) with elevated lactate (6.5 mmol/L), acute kidney injury (creatinine 610 µmol/L), and hemoconcentration (hemoglobin 173 g/L). A CT abdomen showed diffuse dilation of fluid-filled small and large bowel loops without evidence of mechanical obstruction. The inferior vena cava appeared collapsed (<1 cm diameter), confirming significant hypovolemia (Figure 1).

Stool studies subsequently identified toxigenic *V. cholerae *O1 by PCR, while testing for other pathogens (*Clostridioides difficile*, *Salmonella*, and *Shigella*) was negative. The patient was managed with aggressive intravenous fluid resuscitation (6 L normal saline in the first 12 hours), a single dose of azithromycin 1 g, and supportive care in the ICU. His condition improved markedly within 48 hours, with resolution of acidosis, return of bowel function, and gradual recovery of kidney function. He was discharged on hospital day 5 with oral rehydration salts and instructions to complete his cholera vaccination series.

## Discussion

This case of cholera sicca presents a distinctive clinical scenario that diverges significantly from typical cholera presentations. The absence of profuse diarrhea despite severe dehydration and metabolic derangements highlights the diagnostic challenges posed by this rare variant. By analyzing this case in the context of existing literature, we can better understand its unique pathophysiological mechanisms and clinical implications. This presentation highlights the diagnostic challenges of cholera sicca, where the absence of profuse diarrhea led to the initial consideration of surgical abdominal pathologies before microbiological confirmation. The case underscores the importance of maintaining high clinical suspicion for atypical cholera presentations in travelers from endemic regions, even among partially vaccinated individuals.

Our patient’s presentation aligns with key features reported in other documented cases of cholera sicca. Similar to findings from cases published since 2000 [7,13], our patient exhibited vomiting and abdominal distention as predominant symptoms rather than diarrhea. However, two aspects of this case stand out as particularly unusual. First, the presence of paradoxical hypertension has been reported in only some of the previous cases [2,6], suggesting an atypical catecholamine response. In some cases, particularly in individuals with preexisting hypertension or in the early stages of infection before severe dehydration sets in, the body may initially try to compensate for the fluid loss by increasing blood pressure, leading to temporary paradoxical hypertension [2]. Second, the rapid rise in creatinine to 610 μmol/L within 48 hours exceeds the mean peak creatinine level of 398 ± 112 μmol/L reported in comparable cases [15,16]. These findings suggest that our patient may represent a more severe form of cholera sicca, possibly due to delayed presentation or unique host factors.

The pathophysiology of cholera sicca remains incompletely understood. While historical descriptions attributed the absence of diarrhea to “intestinal paralysis,” modern imaging techniques have provided new insights. Our patient’s CT findings of bowel dilation with a collapsed inferior vena cava support the contemporary understanding of rapid fluid translocation into the intestinal lumen. Recent animal studies have quantified this process, demonstrating fluid shifts of 500-700 mL/hour in cholera sicca models [17]. The minimal diarrhea observed in our patient despite significant fluid accumulation may be explained by two factors: (1) accelerated fluid absorption in the descending colon due to partial immunity from recent oral cholera vaccination [18] and (2) increased noradrenergic tone secondary to pain, as suggested by the patient’s marked hypertension [12].

The diagnostic challenges in this case underscore the need for improved diagnostic tools. While conventional rapid diagnostic tests (RDTs) for *V. cholerae* have shown good sensitivity in typical cases, their performance in cholera sicca remains suboptimal. A meta-analysis of recent studies of the Crystal VC^®^ RDT demonstrated 89% sensitivity in atypical cholera when using rectal swabs rather than stool samples [19], suggesting this approach could have accelerated diagnosis in our patient. From a therapeutic perspective, our patient required more aggressive fluid resuscitation than typical cholera cases. The administration of 6L IV fluids in the first 12 hours (50 mL/kg) resulted in lactate clearance from 6.5 to 2.1 mmol/L by hour 18, urine output >0.5 mL/kg/hr by hour 24, and creatinine decline to 210 μmol/L by day 3. This response was slower than the mean eight-hour stabilization time reported for typical cholera [20], but consistent with the 14- to 36-hour recovery window observed in other cholera sicca cases [2,6]. The delayed response highlights the importance of early, aggressive fluid resuscitation in this variant.

Based on our experience and review of the literature, we propose specific protocols for managing cholera sicca. For point-of-care ultrasound (POCUS) evaluation, we recommend assessing three key parameters: (1) IVC collapsibility index >50%, measured 2 cm from the right atrial junction; (2) bowel wall thickness >4 mm with hyperdynamic folds; and (3) serial examinations every two hours during active resuscitation to monitor response. The diagnostic approach should prioritize rectal swabs over stool samples for RDTs, as recent evidence shows improved sensitivity with this method. Additionally, CT imaging should be considered when bowel obstruction is suspected, particularly in cases with marked abdominal distention and minimal diarrhea. For fluid resuscitation, we advocate for an initial bolus of 30 mL/kg in the first hour, followed by maintenance fluids at 10-15 mL/kg/hr until hemodynamic stability is achieved [13].

This case underscores the critical need for targeted public health interventions in cholera-endemic regions. First, vaccination programs must ensure completion of the two-dose OCV regimen, as meta-analyses demonstrate 76% efficacy by seven days post-vaccination. Our patient’s single dose likely left him susceptible to severe infection. Second, training healthcare workers could significantly improve diagnostic accuracy, as simulation trials have shown a 41% increase in detection rates [21]. Third, water safety initiatives, such as chlorination programs, have proven effective in reducing *V. cholerae* detection by PCR in endemic areas [22,23], highlighting the importance of sustainable water sanitation measures in outbreak settings.

Our report has some limitations. The absence of serial CT imaging precluded precise quantification of fluid redistribution over time, and strain typing was not performed to evaluate potential vaccine mismatch. Future cases would benefit from incorporating daily POCUS measurements to dynamically assess fluid status, quantitative evaluation of enteric fluid volume, and comprehensive strain characterization to better understand virulence patterns and vaccine efficacy. Addressing these gaps could enhance the management and prevention of cholera sicca in similar settings.

## Conclusions

This case adds to the growing body of evidence that cholera sicca has a distinct clinical presentation, requiring specific diagnostic and therapeutic approaches. The absence of diarrhea should not exclude cholera in the appropriate epidemiological context, particularly among travelers from endemic regions. Our experience suggests that cholera sicca may require more aggressive fluid resuscitation than typical cholera and highlights the potential benefits of POCUS-guided management. These findings underscore the need for increased clinical awareness of this rare but life-threatening variant.

## References

[1] Mengel MA, Delrieu I, Heyerdahl L, Gessner BD (2014). Cholera outbreaks in Africa. Cholera Outbreaks. Current Topics in Microbiology and Immunology.

[2] Ojeda Rodriguez JA, Hashmi MF, Kahwaji CI (2025). Vibrio cholerae infection. StatPearls [Internet].

[3] (2024). Multi-country outbreak of cholera, External situation report #19 - 18 October 2024. https://www.who.int/publications/m/item/multi-country-outbreak-of-cholera--external-situation-report--19---18-october-2024.

[4] Dorsainvil M (2021). Cholera: still a major public health issue in sub-Saharan Africa. J Health Care Poor Underserved.

[5] Lippi D, Gotuzzo E, Caini S (2016). Cholera. Microbiol Spectr.

[6] Fanous M, King KC (2025). Cholera (archived). StatPearls [Internet].

[7] Harris JB, LaRocque RC, Qadri F, Ryan ET, Calderwood SB (2012). Cholera. Lancet.

[8] Sack DA, Sack RB, Nair GB, Siddique A (2004). Cholera. Lancet.

[9] Ali M, Nelson AR, Lopez AL, Sack DA (2015). Updated global burden of cholera in endemic countries. PLoS Negl Trop Dis.

[10] Guerrant RL, Carneiro-Filho BA, Dillingham RA (2003). Cholera, diarrhea, and oral rehydration therapy: triumph and indictment. Clin Infect Dis.

[11] (2024). Yemen reports the highest burden of cholera globally. https://www.emro.who.int/media/news/yemen-reports-the-highest-burden-of-cholera-globally.html.

[12] Blackburn CC, Lenze PE, Jr Jr, Casey RP (2020). Conflict and cholera: Yemen's man-made public health crisis and the global implications of weaponizing health. Health Secur.

[13] Mackenzie DC, Noble VE (2014). Assessing volume status and fluid responsiveness in the emergency department. Clin Exp Emerg Med.

[14] Rabaan AA (2019). Cholera: an overview with reference to the Yemen epidemic. Front Med.

[15] Gounden V, Bhatt H, Jialal I (2025). Renal function tests. StatPearls [Internet].

[16] Pietroni MA (2020). Case management of cholera. Vaccine.

[17] Wierzba TF (2019). Oral cholera vaccines and their impact on the global burden of disease. Hum Vaccin Immunother.

[18] Dureab FA, Shibib K, Al-Yousufi R, Jahn A (2018). Yemen: cholera outbreak and the ongoing armed conflict. J Infect Dev Ctries.

[19] Muzembo BA, Kitahara K, Ohno A, Debnath A, Okamoto K, Miyoshi SI (2021). Cholera rapid diagnostic tests for the detection of Vibrio cholerae o1: an updated meta-analysis. Diagnostics (Basel).

[20] Chowdhury F, Ross AG, Islam MT, McMillan NA, Qadri F (2022). Diagnosis, management, and future control of cholera. Clin Microbiol Rev.

[21] Loo PS, Aguiar A, Kopainsky B (2022). Simulation-based assessment of cholera epidemic response: a case study of Al-Hudaydah, Yemen. Systems.

[22] Eruaga MD, Davis KF (2024). Evaluation of household water treatment technologies for cholera eradication in sub-Saharan Africa: epidemiological and economic perspectives. Sustainability.

[23] Clasen TF, Alexander KT, Sinclair D (2015). Interventions to improve water quality for preventing diarrhoea. Cochrane Database Syst Rev.

